# Delayed glial clearance of degenerating axons in aged *Drosophila* is due to reduced PI3K/Draper activity

**DOI:** 10.1038/ncomms12871

**Published:** 2016-09-20

**Authors:** Maria D. Purice, Sean D. Speese, Mary A. Logan

**Affiliations:** 1 Jungers Center for Neurosciences Research, Department of Neurology, Oregon Health and Science University, 3181 Southwest Sam Jackson Park Road, Portland, Oregon 97239, USA

## Abstract

Advanced age is the greatest risk factor for neurodegenerative disorders, but the mechanisms that render the senescent brain vulnerable to disease are unclear. Glial immune responses provide neuroprotection in a variety of contexts. Thus, we explored how glial responses to neurodegeneration are altered with age. Here we show that glia–axon phagocytic interactions change dramatically in the aged *Drosophila* brain. Aged glia clear degenerating axons slowly due to low phosphoinositide-3-kinase (PI3K) signalling and, subsequently, reduced expression of the conserved phagocytic receptor Draper/MEGF10. Importantly, boosting PI3K/Draper activity in aged glia significantly reverses slow phagocytic responses. Moreover, several hours post axotomy, early hallmarks of Wallerian degeneration (WD) are delayed in aged flies. We propose that slow clearance of degenerating axons is mechanistically twofold, resulting from deferred initiation of axonal WD and reduced PI3K/Draper-dependent glial phagocytic function. Interventions that boost glial engulfment activity, however, can substantially reverse delayed clearance of damaged neuronal debris.

## Abstract (web summary)

Glial engulfment declines with age, but the mechanism is unclear. Here authors show that in the *Drosophila* olfactory system, glial phagocytosis of injury-induced degenerating axons decreases with age due to reduced PI3K/Draper activity, and restoring Draper in aged glia rescues such defects.

## Graphical abstract

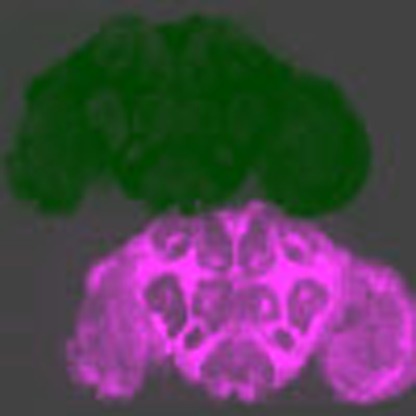

Glial cells are highly sensitive to neuronal stress and damage, and they respond swiftly to trauma by extending membrane projections to injury sites, upregulating essential immune genes and clearing damaged neurons efficiently through phagocytic engulfment. Rapid clearance of dying cells and degenerating neuronal projections thwarts secondary inflammatory responses that can expedite tissue damage and cell death. Given the critical link between glial engulfment activity and neuronal health, it has been proposed that glial phagocytic function may decline with age and, as a result, make the aged brain more sensitive to acute trauma and chronic neurodegenerative conditions^1^,^2^,^3^; however, the intrinsic and extrinsic factors that influence age-dependent changes in glial phagocytic function *in vivo* are still largely unclear.

In aged mice, microglia and astrocytes often display enlarged soma and shorter projections as compared with their young counterparts^4^,^5^,^6^,^7^. These age-dependent changes in morphology suggest that aged glia may be compromised in their ability to sense and/or respond to neuronal stress and degeneration and, in fact, *in vitro* studies have suggested that microglia harvested from older animals display defective phagocytic activity^8^. Although there is growing evidence that the transcriptional profile of glia changes with age^9^,^10^, few *in vivo* functional studies have pinpointed specific glial factors that contribute to altered engulfment activity and typical age-related decline in neuroprotection.

Studies in *Drosophila* have provided critical insight into the molecular, biochemical and systemic changes that govern ageing and longevity. For example, reduced insulin-like signalling, mitochondrial fatigue, defects in autophagy and altered transcription/translation have all been confirmed to influence senescence across species^11^,^12^,^13^. Thus, *Drosophila* offers a well-established genetic model system to rapidly interrogate the molecular intricacies of ageing physiology and identify specific pathways that can be targeted to delay age-related decline and extend lifespan. Here we use an acute axotomy assay in the olfactory system of adult *Drosophila* to investigate how ageing alters glial responses to axon degeneration *in vivo*. We demonstrate that glial clearance of damaged axons is significantly delayed in aged animals due to an age-dependent decline in translation of the critical glial recognition engulfment receptor Draper, which results from reduced phosphoinositide-3-kinase (PI3K) signalling. Importantly, in aged glia, Draper levels fall below a critical threshold required to activate a Draper-dependent STAT92E transcriptional programme in local glia in response to axon injury.

## Results

### Clearance of severed axons is delayed in aged *Drosophila*

**Figure 1:**
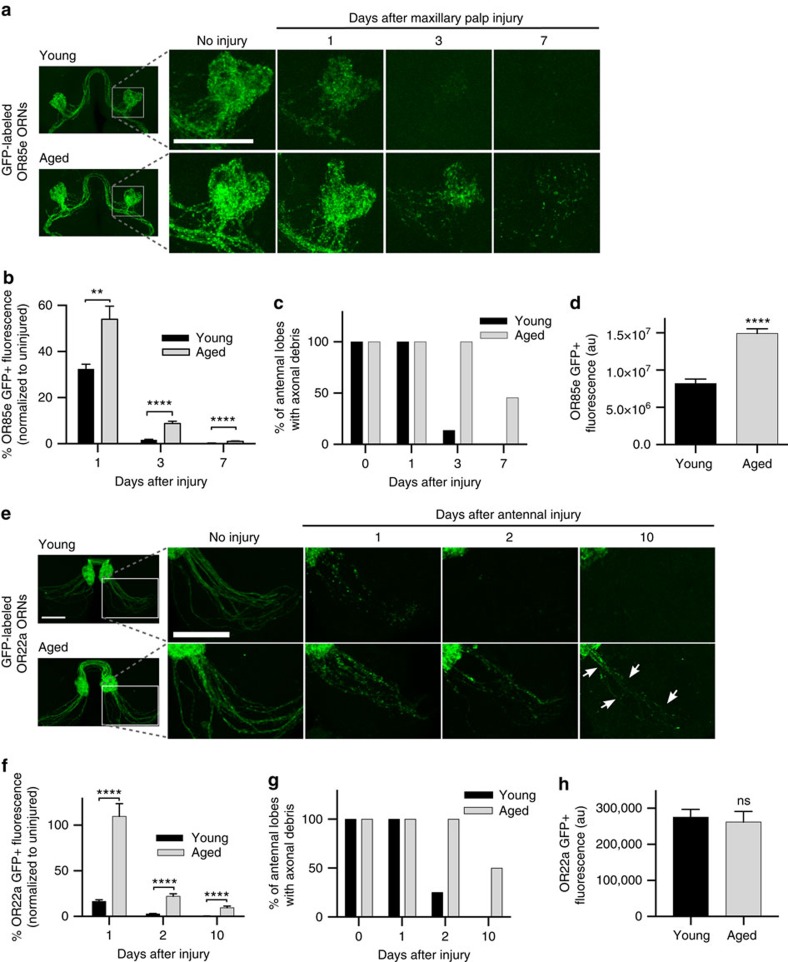
Aged glia fail to efficiently clear degenerating axons in the adult *Drosophila* brain. (**a**) GFP-labelled maxillary ORN axons before and after maxillary nerve axotomy in young and aged flies. Zoomed images show OR85e-innervated glomeruli. (**b**) Quantification of GFP+ debris in OR85e glomeruli; mean±s.e.m. plotted; **P<0.01 and ****P<0.0001, Mann–Whitney *post-hoc* test. (**c**) Percentage of antennal lobes containing visible GFP+ OR85e debris after maxillary nerve axotomy. (**d**) Total GFP fluorescence in OR85e glomeruli; mean±s.e.m. plotted; ****P<0.0001, unpaired *t*-test. (**e**) GFP-labelled antennal ORN axon projections before and after antennal nerve axotomy in young and aged flies. GFP+ axonal material was still present in aged brains 10 days after antennal nerve injury (white arrows). (**f**) Quantification of OR22a axonal debris in **e**; mean±s.e.m. plotted; ****P<0.0001, Mann–Whitney *post-hoc* test. (**g**) Percentage of antennal lobes containing visible tracts of antennal GFP^+^ axons. (**h**) Total GFP fluorescence of OR22a axons; mean±s.e.m. plotted; NS, not significant, unpaired *t*-test. *N*≥20. Scale bar, 30 μm. Representative confocal *Z*-stacks shown for all panels in **a** and **e**. Genotypes: **a**–**d**: *w*^*1118*^*;OR85e-mCD8::GFP/+*; **e**–**h**: *w*^*1118*^*;OR22a-Gal4, UAS mCD8::GFP/+.*

We used an acute axotomy assay in the olfactory system of adult flies to explore how ageing influences glial responses to axon degeneration *in vivo.* Bilateral removal of the third antennal segments or maxillary palps triggers Wallerian degeneration (WD) of olfactory receptor neuron (ORN) axons that project into the antennal lobes of the central brain^14^,^15^. Local ensheathing glia extend membranes to accumulate on degenerating axons, upregulate key immune factors and efficiently clear axonal debris through phagocytic engulfment^15^,^16^,^17^,^18^,^19^. We compared glial clearance of degenerating ORN axons in young and aged flies by severing the maxillary nerves of flies expressing membrane-tethered green fluorescent protein (GFP) in a subset of maxillary palp ORNs (*OR85e-mCD8::GFP*) and quantifying GFP^+^ axonal debris in OR85e-innervated glomeruli at various time points post axotomy. Clearance of GFP-labelled axon material was significantly delayed in aged animals after injury (Fig. 1a–c). We observed higher basal levels of GFP in the OR85e glomeruli of aged *OR85e-mCD8::GFP* animals (Fig. 1d). Thus, we took an alternative approach to label ORN axons and ensure that age-dependent changes in axonal GFP levels did not influence clearance post axotomy. Specifically, we used the robust GAL4/UAS system to express membrane-tethered GFP in a subset of ORNs (*OR22a-Gal4, UAS-mCD8::GFP*), which resulted in comparable basal GFP levels in young and aged neurons (Fig. 1h and Supplementary Fig. 1), and then quantified clearance of GFP^+^ axonal debris after antennal nerve axotomy. Again, we observed significantly more GFP^+^ axonal debris lingering in the brains of aged flies at each time point (Fig. 1e–g and Supplementary Fig. 1). For example, 10 days post injury, identifiable tracts of fragmented axons were visible in almost half of the aged flies, whereas no tracts were detectable in young brains (Fig. 1g). Delayed removal of OR22a GFP^+^ material in aged animals, despite the fact that GFP levels are comparable in uninjured young and aged flies, suggests that differences in OR22a axonal debris clearance are not simply due to the fact that glia must manage a higher axonal GFP load in aged brains.

### Glia are not recruited to aged degenerating axons

Efficient clearance of degenerating olfactory axons requires that local ensheathing glia expand their membranes to accumulate on actively degenerating nerves^15^,^17^,^18^,^19^. We used flies that expressed glial membrane-tethered GFP (*repo-Gal4, UAS-mCD8::GFP*) to quantify recruitment of glial membranes to injured maxillary ORN axons 24 h after maxillary nerve injury and found that accumulation of glial membranes was significantly reduced in aged flies (maxillary injury; Supplementary Fig. 2a,b). Similarly, expansion of ensheathing glial membranes around the antennal lobes was attenuated after severing the antennal nerves in aged animals (antennal injury; Supplementary Fig. 2a,c). We also immunostained aged and young brains for the pan-glial marker Repo and, consistent with a previous study^20^, found no reduction in the number of glial cells in the central brain regions adjacent to antennal lobes (Supplementary Fig. 3), suggesting that poor glial membrane recruitment and phagocytic clearance of damaged axons in the aged brain is not a result of glial cell death.

### Draper is reduced in aged *Drosophila* glia

**Figure 2:**
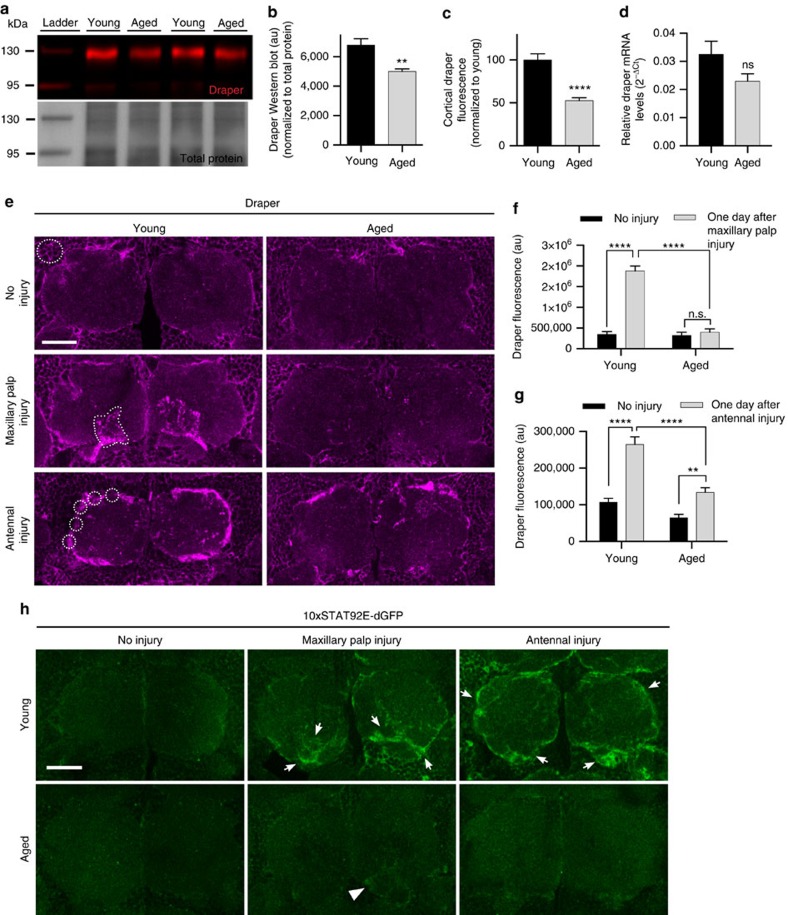
Axotomy-induced activation of Stat92E and upregulation of Draper decline with age. (**a**) Western blotting with α-Draper (red, top panel) and MemCode general protein stain (bottom panel) of head lysates from young and aged flies. (**b**) Quantification of Draper western blotting. **P<0.01, unpaired *t*-test. (**c**) Quantification of Draper fluorescence in the cortex of young and aged brains. Representative region of interest (ROI) in young uninjured panel in **e**; mean±s.e.m. plotted; ****P<0.0001, unpaired *t*-test. (**d**) Quantitative PCR of *Draper-I* transcript levels in young and aged brains; mean±s.e.m. plotted, young *N*=9, aged *N*=10, unpaired *t*-test. (**e**) Representative single confocal slices of Draper immunostained brains. White dotted outlines on injured images show ROIs used for quantification of Draper in **c**,**f** and **g**. (**f**) Quantification of Draper fluorescence in OR85e maxillary palp glomeruli before and after maxillary nerve injury; *N*≥22; mean±s.e.m. plotted; **P<0.01 and ****P<0.0001. Two-way analysis of variance (ANOVA) with Sidak *post-hoc* test. (**g**) Quantification of Draper fluorescence in antennal lobe ensheathing glia before and after antennal nerve injury; *N*≥22. mean±s.e.m. plotted; **P<0.01 and ****P<0.0001. Two-way ANOVA with Sidak *post hoc* test. (**h**) Stat92E-dependent activation of dGFP in the antennal lobe region. Robust reporter activity is observed in young flies 24 h after maxillary or antennal nerve injury (arrows) but virtually undetectable in aged animals (arrowhead). Scale bars, 30 μm. Genotypes: **a**–**g**: *w*^*1118*^. **h**: *10xStat92E-dGFP/+*; NS, not significant; au, arbitrary units.

Draper/MEGF10 is a highly conserved phagocytic receptor required for glial engulfment of apoptotic cells and pruned neural projections during development^16^,^21^,^22^,^23^,^24^ and is essential for glial clearance of adult degenerating axons^15^,^16^,^17^,^18^,^19^,^25^. Thus, we asked how glial Draper expression and/or function might change with age. Indeed, western blotting (Fig. 2a,b) and immunostaining (Fig. 2c and top panels in Fig. 2e) revealed that Draper protein levels were significantly reduced in the aged central nervous system, although *draper-I* transcript levels were unchanged (Fig. 2d). Importantly, we found that injury-induced upregulation of Draper protein in local ensheathing glia was also attenuated. In young flies, Draper accumulates on maxillary palp-innervated glomeruli within 24 h after maxillary nerve axotomy^15^,^17^,^18^,^19^, but we found this response to be virtually undetectable in aged brains (middle panels in Fig. 2e,f). Even after eliciting a substantially larger injury by severing the majority of ORNs by antennal nerve axotomy, Draper upregulation was still significantly reduced in aged ensheathing glia (lower panels in Fig. 2e,g).

### Activation of glial STAT92E is attenuated in aged glia

Recent work has shown that following olfactory nerve axotomy in young animals, the STAT92E transcription factor is activated in local ensheathing glia in a Draper-dependent manner to boost *draper-I* transcript levels^26^. This positive autoregulatory feedback loop ensures that Draper levels are sufficiently increased when the glial phagocytic load is high following axon degeneration^26^. Thus, we hypothesized that STAT92E activity may be attenuated in aged animals after axon injury. The *in vivo* transcriptional reporter *10XSTAT92E-dGFP*, which contains ten tandem STAT92E consensus binding sites controlling expression of destabilized GFP (dGFP), serves as a reliable readout of ensheathing glial STAT92E activity before and after axon injury^26^. We found that induction of dGFP after maxillary or antennal nerve injury on aged *10XSTAT92E-dGFP* flies was almost undetectable 24 h after injury, in contrast to young animals (Fig. 2h), suggesting that weak upregulation of Draper in aged glia following axotomy may result from reduced activation of STAT92E-dependent transcriptional cascades.

### Clearance of injured axons is delayed in draper heterozygotes

**Figure 3:**
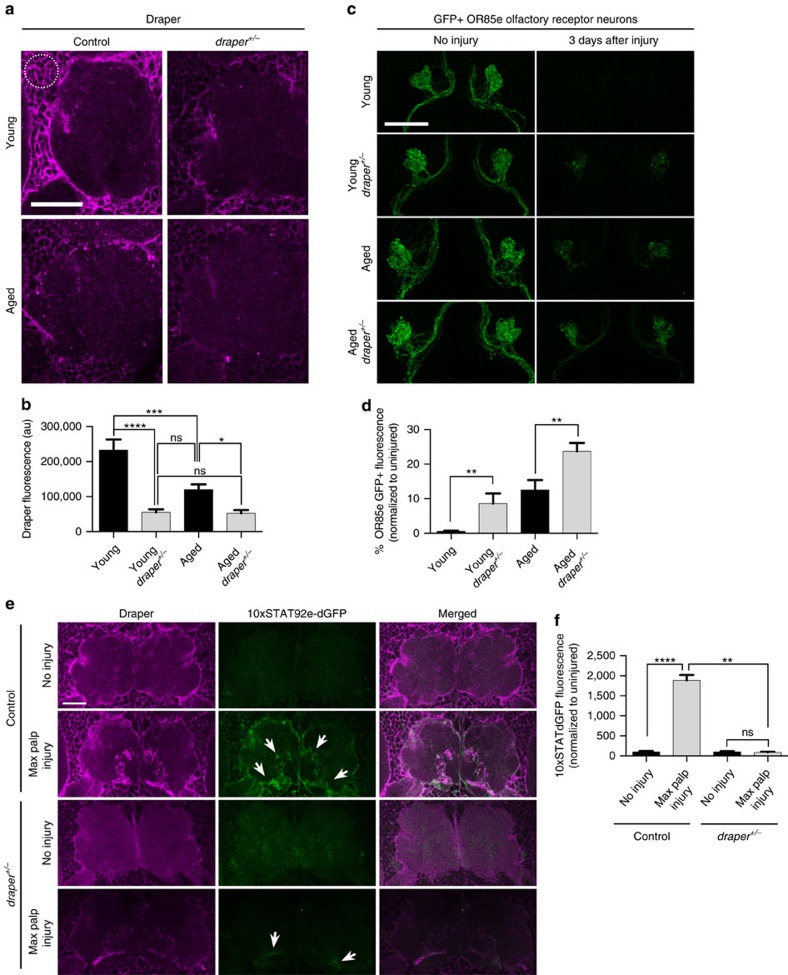
Glial responses to degenerating axons are attenuated in *draper* heterozygotes. (**a**) Representative single confocal slices of Draper immunostained brains: young (*OR85e-mCD8::GFP*), young draper^+/*−*^ (*OR85e-mCD8::GFP; draper*^*Δ5*^*/+*), aged (*OR85e-mCD8::GFP*), aged draper^+/*−*^ (*OR85e-mCD8::GFP; draper*^*Δ5*^*/+*). White dotted outline indicates region of interest (ROI) used for quantification of basal Draper in **b**. (**b**) Quantification of cortical Draper immunostainings shown in **a**; mean±s.e.m. plotted; NS, not significant, *P<0.05, ***P<0.001 and ****P<0.0001, one-way analysis of variance (ANOVA) with Sidak *post hoc* test; *N*≥16. (**c**) Representative confocal projections of GFP-labelled OR85e axons. (**d**) Quantification of axon clearance in **c**; mean±s.e.m. plotted; **P<0.01; unpaired *t* test; *N*≥14. (**e**) Single confocal images of antennal lobe regions showing STAT92E-dependent activation of dGFP (green) and Draper (magenta) in Control (*10xSTAT92E-dGFP/+)* and draper^+/*−*^ (*10xSTAT92E-dGFP/+; draper*^*Δ5*^*/+*) animals. Arrows show expected upregulated of *10xSTAT92E-dGFP* reporter activity after injury. (**f**) Quantification of dGFP on maxillary glomeruli shown in **e**; mean±s.e.m. plotted; NS, not significant, **P<0.01 and ****P<0.0001, one-way ANOVA with Sidak *post-hoc* test; *N*≥18. Scale bars, 30 μm.

Notably, basal Draper levels are significantly reduced in young Draper heterozygous (*draper*^*+/−*^) flies and, in fact, are comparable to the levels in aged *draper*^*+/+*^ brains (Fig. 3a,b). We compared clearance of degenerating OR85e axons in young and aged *draper*^*+/+*^ and *draper*^*+/−*^ adults. Strikingly, the clearance defect of young *draper*^*+/−*^ and aged *draper*^*+/+*^ animals were similar, and loss of one copy of draper further exacerbated this phenotype in aged animals (Fig. 3c,d). Finally, we compared activation of *10XSTAT92E-dGFP* reporter activity in young and aged animals following maxillary nerve axotomy and, surprisingly, detected no increase in *10XSTAT92E-dGFP* reporter activity in young animals that lacked one copy of *draper* (Fig. 3e,f). These results indicate that loss of Draper in adult glia below a critical threshold can significantly compromise downstream immune responses, including injury-responsive STAT92E transcriptional cascades and phagocytic function.

### Draper reverses axotomy response phenotypes in aged animals

**Figure 4:**
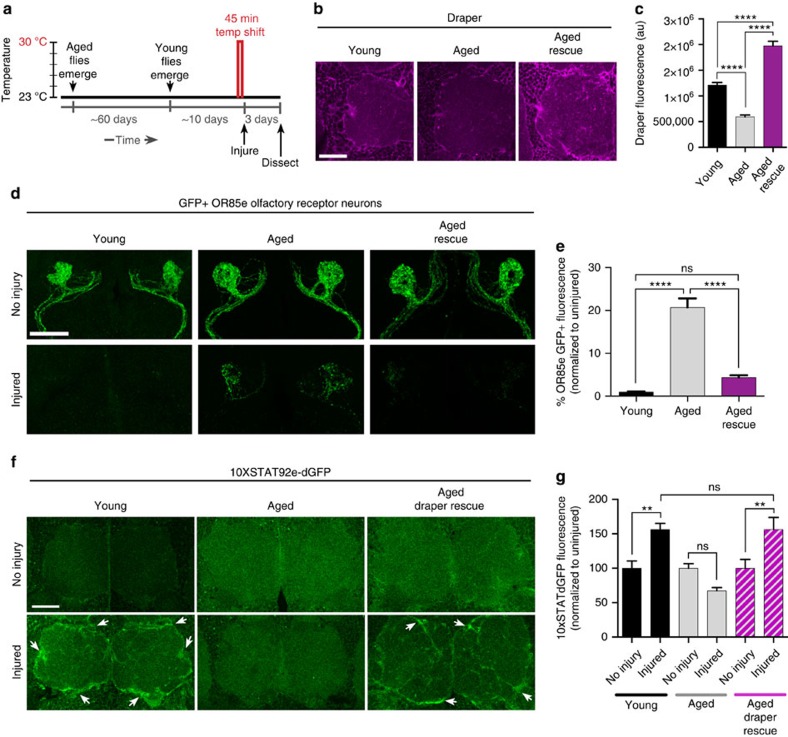
Expression of Draper in aged glia rescues glial clearance of axonal debris. (**a**) Schematic of the Gal80^ts^ temperature-regulated paradigm to acutely induce expression of Draper-I in aged glia after injury. (**b**) Representative images of Draper (magenta) immunostained brains. (**c**) Quantification of Draper fluorescence in the cortex of Young, Aged and Aged Draper-I rescue brains; mean±s.e.m. plotted; ****P<0.0001, one-way analysis of variance (ANOVA) with Sidak *post-hoc* test; *N*≥20. (**d**) Confocal projections of OR85e GFP^+^ axonal projections in uninjured flies versus 3 days after maxillary nerve axotomy. Expression of Draper-I in aged glia (Aged rescue) restored normal clearance of axonal debris. (**e**) Quantification of axonal clearance shown in **d**; mean±s.e.m. plotted, NS, not significant, ****P<0.0001, one-way ANOVA with Sidak *post-hoc* test. *N*≥14. (**f**) Representative images of antennal lobes showing expression of dGFP under the control STAT92E activity (*10XSTAT92E-dGFP*) immunostaining (green) in Young, Aged and Aged Draper-I rescue animals. (**g**) Quantification of dGFP shown in **f**; mean±s.e.m. plotted, NS, not significant, **P<0.01, one-way ANOVA with Sidak *post-hoc* test; *N*≥18. NS, not significant. Scale bars, 30 μm. Genotypes: **b**–**e**, Young=*w*^*1118*^*;OR85e-mCD8::GFP, tubulin-Gal80*^*ts*^*/+; repo-Gal4/+*. Aged=*w*^*1118*^*;OR85emCD8:: GFP, tubulin-Gal80ts/+; repo-Gal4/+*. Aged rescue=*w*^*1118*^*;OR85e-mCD8::GFP, tubulin-Gal80ts/UAS-Draper-I; repo-Gal4/+*. **f**,**g**, Young=*w*^*1118*^*; 10xSTAT92e-dGFP, tubulin-Gal80ts/+; repo-Gal4/+.* Aged=*w*^*1118*^*; 10xSTAT92e-dGFP, tubulin-Gal80ts/+; repo-Gal4/+*. Aged Draper rescue=*w*^*1118*^*; 10xSTAT92e-dGFP, tubulin-Gal80ts/ UAS-Draper-I; repo-Gal4/+*.

As maintaining proper Draper levels is essential for efficient glial clearance of degenerating axons, we reasoned that restoring Draper in aged glia might rescue engulfment phenotypes in older animals. We used a temperature-sensitive version of Gal80 (Gal80^ts^)^27^ to temporally control activation of a *UAS-Draper-I* transgene and provide a short pulse of Draper expression in aged glia immediately following maxillary nerve axotomy (Fig. 4a). This paradigm increased Draper levels in aged glia at both the protein (Fig. 4b,c) and transcript level (Supplementary Fig. 4a). Strikingly, re-expression of Draper in aged glia (Aged rescue) restored phagocytic clearance of degenerating OR85e axons 3 days after axon injury (Fig. 4d,e) and also rescued injury-induced activation of *10XSTAT92E-dGFP* in aged animals (Fig. 4f,g). We should note that modestly raising Draper levels in young glia above endogenous levels does not result in faster glial clearance and, in fact, forcing higher levels of Draper expression can ultimately inhibit glial clearance of axonal debris^16^ (Supplementary Fig. 4). Our results suggest that loss of the Draper receptor is a key causal factor in delayed glial responses to degenerating axons in the aged brain.

### PI3K reverses axotomy response phenotypes in aged animals

**Figure 5:**
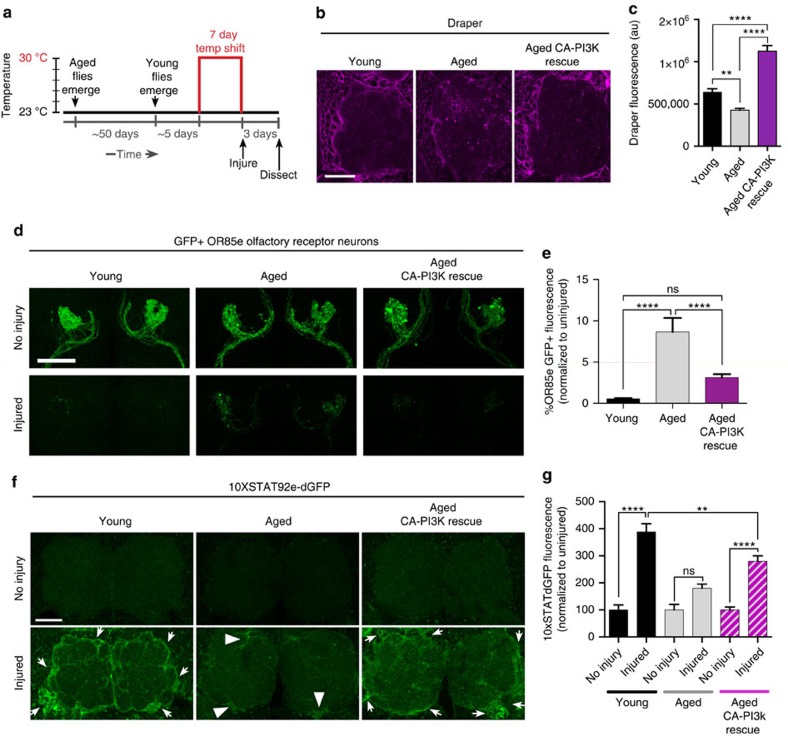
Expression of CA-PI3K in aged glia rescues glial clearance of axonal debris. (**a**) Schematic of experimental paradigm to induce expression of constitutively active-PI3K (CA-PI3K) in aged glia. (**b**) Representative Draper immunostainings of Young, Aged and Aged CA-PI3K rescue brains. (**c**) Quantification of Draper fluorescence in the cortex of Young, Aged and Aged CA-PI3K rescue brains shown in **b**. mean±s.e.m. plotted; **P<0.01 and ****P<0.0001, one-way analysis of variance (ANOVA) with Sidak *post-hoc* test; *N*≥26. (**d**) GFP^+^ OR85e axons before and 3 days post axotomy. Representative *Z*-stack projections shown. (**e**) Quantification of GFP^+^ axonal debris in **d**; mean±s.e.m. plotted; NS, not significant, ****P<0.0001, one-way ANOVA with Sidak *post-hoc* test, *N*≥20. (**f**) Representative images of antennal lobes showing expression of dGFP under the control STAT92E activity (*10XSTAT92E-dGFP*) immunostaining (green) in Young, Aged and Aged CA-PI3K rescue. (**g**) Quantification of dGFP shown in **f**. NS, not significant, **P<0.01 and ****P<0.0001, one-way ANOVA with Sidak *post-hoc* test; *N*≥18. NS, not significant. Scale bars, 30 μm. Genotypes: **b**–**e**, Young=*w*^*1118*^*;OR85e-mCD8::GFP, tubulin-Gal80ts/+; repo-Gal4/+*. Aged=*w*^*1118*^*;OR85e-mCD8::GFP, tubulin-Gal80ts/+; repo-Gal4/+*. Aged CA-PI3K rescue=*UAS-PI3K92eCAAX;OR85e-mCD8::GFP, tubulin-Gal80ts/+; repo-Gal4/+*. **f**,**g**, Young=*w*^*1118*^*;10xSTAT92e-dGFP, tubulin-Gal80ts/+; repo-Gal4/+*. Aged=*w*^*1118*^*; 10xSTAT92e-dGFP, tubulin-Gal80ts/+; repo-Gal4/+*. Aged CA-PI3K rescue=*UAS-PI3K92eCAAX; 10xSTAT92e-dGFP, tubulin-Gal80ts/+; repo-Gal4/+*.

In young animals, PI3K92E is required for proper basal Draper expression in glial cells and for timely clearance of degenerating ORN axonal debris^26^, and glial overexpression of a well-characterized constitutively active version of PI3K92E (CA-PI3K) (*UAS- caPi3K92E*^*caax*^)^28^,^29^,^30^ in young flies increases Draper levels^26^. We expressed this CA-PI3K in the glial cells of young flies that lacked one copy of draper (*draper*^*+/−*^) and found that this was sufficient to rescue delayed phagocytic clearance of OR85e axonal debris 3 days after axotomy (Supplementary Fig. 5a,b) and also significantly raised basal Draper levels in the heterozygous background (Supplementary Fig. 5c,d). Thus, we hypothesized that age-dependent decline in PI3K92E signalling may account for loss of Draper and delayed glial responses to degenerating axons in older animals. We expressed CA-PI3K acutely in aged glial cells (Fig. 5a). Raising PI3K activity restored basal Draper levels (Fig. 5b,c) and also rescued delayed clearance of degenerating OR85e axons (Fig. 5d,e). Basal levels of OR85e-mCD8::GFP are higher in aged animals (Fig. 1d). Thus, we cannot rule out the possibility that this may partially contribute to the differential clearance of GFP-labelled OR85e material in young versus aged brains. Nonetheless, our findings indicate that age-dependent changes in Draper expression and downstream signalling pathways contribute to delayed destruction of severed axons in older animals.

Next, we assessed STAT92E activity in aged flies following expression of CA-PI3K by monitoring *10XSTAT92E-dGFP* induction 1 day after antennal nerve axotomy. Although we do not detect an increase in dGFP in aged glia post injury (Fig. 5f,g, Aged), dGFP levels are significantly increased when PI3K signalling is constitutively activated (Fig. 5f,g, Aged CA-PI3K rescue). Together, these findings suggest that, with age, reduced PI3K activity causes Draper to fall below a critical threshold required for ensheathing glia to activate a STAT92E-dependent transcriptional programme post injury, which ensures adequate upregulation of engulfment genes and efficient clearance of neuronal debris.

### TOR is required for glial Draper and severed axon clearance

**Figure 6:**
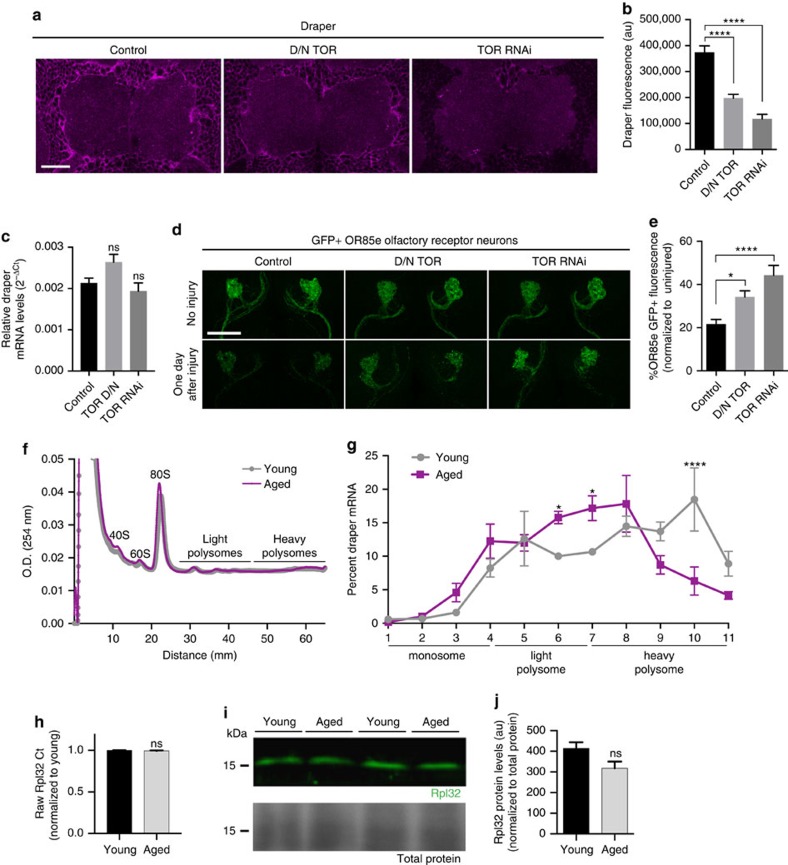
Inhibition of TOR in adult glia inhibits Draper and glial clearance of axon debris in young animals. (**a**) Single confocal slices of antennal lobe regions. Brains immunostained with Draper. (**b**) Quantification of cortical Draper immunostainings shown in **a**; mean±s.e.m. plotted; ****P<0.0001, one-way analysis of variance (ANOVA) with Sidak *post-hoc* test; *N*≥16. (**c**) Quantitative PCR (qPCR) analysis of *draper-I* transcript levels in adult heads; mean±s.e.m. plotted, NS, not significant, one-way ANOVA with Sidak *post-hoc* test; *N*=3 biological replicates. (**d**) Representative *Z*-stack projections of OR85e GFP^+^ axons. (**e**) Quantification of GFP^+^ axonal debris shown in **d**; mean±s.e.m. plotted; *P<0.05 and ****P<0.0001, one-way ANOVA with Sidak *post-hoc* test. *N*=22. (**f**) Polysome profiles for young and aged whole head lysates resolved on a 10–60% sucrose gradient. Absorbance continuously monitored at 254 nm during fractionation shown. (**g**) *draper-I* mRNA in each fraction was quantified by qPCR and normalized to the housekeeping gene *Rpl32* and *luciferase* (control for RNA recovery). mean±s.e.m. plotted; *P<0.05 and ****P<0.0001, two-way ANOVA with uncorrected Fisher's LSD *post-hoc* test. *N*=3 groups of 30 w^118^ fly heads/age. (**h**) Normalized *Rpl32* Ct values pooled from four experiments on young and aged w^118^ brain lysates; mean±s.e.m. plotted, NS, not significant, unpaired *t*-test. *N*=17 biological replicates/age. (**i**) Representative western blotting for Rpl32 (green) and MemCode total protein stain (bottom panel) performed on head lysates from young and aged flies. (**j**) Quantification of Rpl32 western blottings; unpaired *t*-test. *N*=4 biological replicates/age. Genotypes: **a**–**e**, Control=*w*^*1118*^*;OR85e-mCD8::GFP, tubulin-Gal80ts/+; repo-Gal4/+*. D/N TOR=*w*^*1118*^*;OR85e-mCD8::GFP, tubulin-Gal80ts/+; repo-Gal4/UAS-D/N TOR*. TOR RNAi=*w*^*1118*^*;OR85e-mCD8::GFP, tubulin-Gal80ts/+; repo-Gal4/UAS-TOR*^*RNAi*^. **f**–**j**, *w*^*1118*^. Scale bars=30 um.

To determine whether there is an age-dependent basal reduction in *draper* at the transcriptional level, we compared transcript levels of Draper-I in young versus aged brains by quantitative real-time PCR and detected no significant difference (Fig. 2d). Thus, we reasoned that reduced translation might account for reduced Draper protein levels in aged brains, in particular as PI3K is a well-known positive regulator of target of rapamycin (TOR)-mediated translation^31^,^32^,^33^ and glial knockdown of PI3K92E does not alter *draper-I* transcript levels in young flies^26^. We performed glial-specific knockdown of TOR (*UAS-TOR*^*RNAi*^) or expressed a dominant-negative version of TOR (*UAS-D/N TOR*) in young flies and found that in both instances Draper levels were reduced (Fig. 6a,b) and glial clearance of severed OR85e axons was significantly delayed (Fig. 6d,e). Notably, although Draper protein levels were lowered by inhibiting the expression/function of TOR, *draper-I* transcript levels were unchanged (Fig. 6c). These findings support the notion that loss of Draper following inhibition of PI3K results from a TOR-dependent decline in translation.

### Draper translation is reduced in aged brains

To compare Draper translation in young versus aged brains, we analysed the polysome load of the *draper-I* transcript by sucrose gradient centrifugation. Polysome profiles from young and aged brain lysates were recorded and fractionated (Fig. 6f), and we performed quantitative PCR to determine *draper-I* transcript levels in each fraction. In aged brains, we observed a reduction in heavy polysomal-associated *draper-I* messenger RNA and a corresponding increase in the light polysome fraction, as compared with the housekeeping gene *Rpl32* (Fig. 6g), which indicates reduced translation initiation of *draper-I* in aged animals. Transcript and protein levels of our housekeeping molecule Rpl32 did not significantly change with age (Fig. 6h–j). Although TOR controls global protein synthesis, transcripts that contain contain 5′-terminal oligopyrimidine (TOP) motifs are particularly sensitive to TOR regulation^34^,^35^,^36^. We performed a computational analysis of the draper-I mRNA sequence using the RegRNA (Regulatory RNA Motifs and Elements Finder) programme^37^ and found that the draper-I transcript contains three TOP domains in the 5′-untranslated region. It remains to be determine how each of these TOP motifs contribute to age-dependent changes in Draper-I translation but, interestingly, translational activation of TOP-containing mRNAs is highly dependent on PI3K activity^35^,^36^. Together, our findings suggest that, with age, draper gene transcription is unaltered but Draper receptor levels are attenuated due to reduced translation rates, likely through inhibition of PI3K/TOR function.

### Axonal fragmentation is delayed post axotomy in aged animals

**Figure 7:**
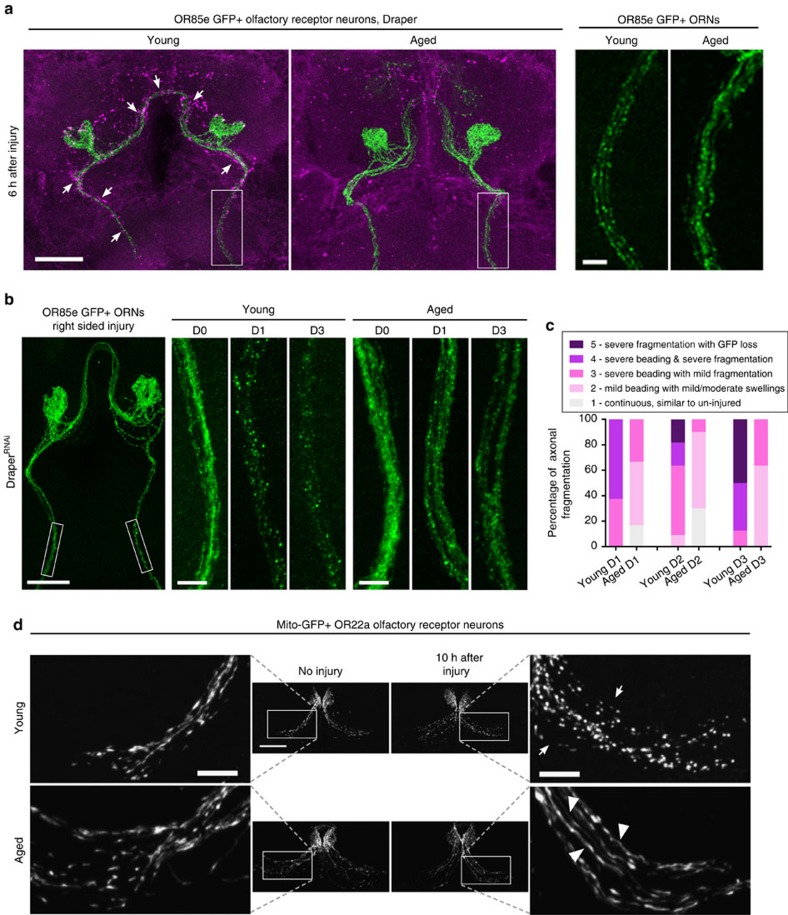
WD of severed ORNs is delayed in aged animals. (**a**) Representative *Z*-stack projections shown from young and aged brains 6 h post-axotomy. Draper (magenta) accumulation is visible on young axons at this time point (white arrows). Scale bar, 30 μm. Zoomed images of axons (right) are from white, boxed regions. Scale bar, 10 μm. (**b**) Representative images of young and aged axons expressing membrane-tethered GFP from uninjured (D0) animals and 1 day (D1) or 3 days (D3) after injury in glial Draper-depleted flies. Scale bar, 30 μm in the left panel and 5 μm in higher magnification panels. (**c**) Blind scoring analysis of fragmentation status of young and aged axons 1 day (D1), 2 days (D2) or 3 days (D3) post-axotomy shown in **b**. (**d**) Representative projections of young and aged flies expressing mito-GFP in a subset of olfactory axons before and 10 h after axotomy. mito-GFP pattern appeared punctate after injury in young animals (white arrows) but continuous in aged axons (white arrowheads). Scale bar, 30 μm in central panels and 10 μm for magnified images. Genotypes: **a**, *w*^*1118*^*; OR85emCD8:: GFP/+.* **b**,**c**, *w*^*1118*^*; OR85emCD8:: GFP/+; repo-Gal4/UAS-Draper*^*RNAi*^. **d**, *OR22a-Gal4/UAS-mito-HA-GFP*.

In aged mice, crushed or severed peripheral nerves fragment more slowly as compared with young animals^38^,^39^. Therefore, we wondered whether *Drosophila* olfactory nerves are similarly delayed in initiating a WD programme in aged flies and, potentially, in releasing signals that elicit innate responses from local ensheathing glia within the first ∼24 h post injury. First, we compared fragmentation rates of young and aged GFP-labelled OR85e axons 6 h after maxillary nerve axotomy. Fragmentation did appear to be more pronounced in young axons at this time point (Fig. 7a); however, we also detected more glial infiltration into the glomeruli (denoted by Draper) at this early time point in young flies (white arrows, Fig. 7a). We were concerned that faster glial recruitment of young glia would obscure our ability to detect differences in intrinsic axon fragmentation rates in young versus aged animals over an extended time course. Therefore, we examined axon fragmentation rates in flies expressing *UAS-Draper*^*RNAi*^ under the control of *repo-Gal4*, to ensure that recruitment/infiltration of glial cells after axon injury was blocked^15^. Using this strategy, we compared fragmentation rates of young and aged OR85e axons. We applied a blind scoring analysis to images of axons 0, 24, 48 and 72 h post-maxillary nerve injury, to assess the extent of axonal beading and fragmentation over a fixed distance, and found that aged axons appeared to fragment more slowly (Fig. 7b,c). For example, 72 h post injury (the last time point analysed), continuous axonal projections could be traced in >50% of aged brains, whereas no continuous projections were visible in young brains (Fig. 7c). Next, we compared mitochondrial morphology in young and aged axons post injury, as mitochondria undergo rapid fragmentation after axotomy and, in fact, are active factors in driving WD^40^,^41^,^42^,^43^. We genetically expressed mitochondrial targeted GFP (*UAS-mito::GFP*) in a subset of ORNs (*OR22a-Gal4*). Mito-GFP patterns appeared similar in young and aged uninjured animals, and included both discrete puncta and continuous tubular structures, which represent ongoing mitochondrial fission and fusion (no injury, Fig. 7d). Ten hours after severing antennal nerves, the mito-GFP signal became highly punctate in young axons (arrows, Fig. 7d). Conversely, in aged axons, mito-GFP distribution appeared more tubular, with fewer discrete puncta, even compared with aged uninjured axons (arrowheads, Fig. 7d). Together, these findings suggest that initiation of WD after axotomy is qualitatively different and/or slowed in aged flies. Delayed axon-to-glia signalling after injury in aged brains may explain why Draper is not sufficient to reverse glial clearance defects in the first 24 h post axotomy.

## Discussion

Our studies provide new mechanistic insight into how ageing alters glial–axon interactions and glial responses to neural injury. We provide direct *in vivo* evidence that dysfunctional glial engulfment in aged *Drosophila* is largely due to downregulation of the Draper receptor at the protein level as a result of decreased PI3K92E activity and translation efficiency. Our findings are consistent with previous reports from other species that reduced translation and/or protein degradation, as opposed to reduced transcriptional activity, is an important feature of ageing coupled to declining cellular and organismal function^44^,^45^,^46^. Here we show that forced activation of PI3K signalling rescues both reduced Draper expression and delayed glial clearance of severed axons several days after axotomy, which implicates PI3K-dependent signalling as a critical age-sensitive cascade that strongly influences glial responses to axon degeneration. Importantly, upregulation of glial Draper after axon injury also significantly rescues glial clearance defects in aged animals. Thus, although many glial proteins are undoubtedly affected by age-dependent decline in translation/stability, we propose that loss of the Draper receptor specifically inhibits the engulfment activity of aged glia, which is a critical neuroprotective feature of glia.

Draper is essential for glial membrane expansion/hypertrophy following axotomy and subsequent phagocytic removal of damaged axons in adult flies^15^,^16^,^17^,^18^,^19^. Our results are consistent with reports of diminished injury-induced motility of vertebrate microglia and *in vitro* experiments that suggest aged microglia have reduced phagocytic capacity^4^,^5^,^6^,^7^,^8^,^47^. Therefore, our findings may be extrapolated to mammals, as Draper and the mammalian homologue MEGF10/Jedi signal through highly conserved tyrosine kinase cascades and are required for glial engulfment of degenerating axons, synapses and/or apoptotic neurons in a variety of contexts^15^,^18^,^19^,^21^,^22^,^23^,^24^,^48^,^49^. Moreover, recent work has shown that *Drosophila* glia can internalize pathogenic human Huntingtin protein^50^ and neurotoxic amyloid-β42 peptides (A. Ray and M.A. Logan, unpublished) in a Draper-dependent manner, which further bolsters the notion that age-related decreases in engulfment activity are coupled to an increased risk for age-related neurodegenerative disorders, including Alzheimer's disease^51^,^52^. Finally, loss of Draper results in shortened lifespan and increased risk for neuronal apoptotic death in adult *Drosophila*^53^,^54^. Together, this body of work highlights the Draper/MEGF10 pathway as an exciting new therapeutic candidate to boost innate glial immune activity, including phagocytosis, to enhance neuroprotection with advanced age.

Recent work from Doherty *et al*.^26^ identified PI3K92E as a positive regulator of Draper in young glia and also showed that STAT92E was required to upregulate *draper-I* in young glia responding to injury. Importantly, our work reveals that PI3K-dependent reduction of Draper translation is a key limiting factor responsible for delayed phagocytic responses to neural injury in the aged brain. Stimulating PI3K activity and/or raising Draper levels largely rescues poor clearance of degenerating axons and defects in Draper/STAT92E-dependent transcriptional activity that are typically observed in aged animals. These results indicate that despite the many fundamental biological shifts that occur in the ageing brain, loss of PI3K/Draper is a primary reason for declining innate glial immune function in senescent animals. Moreover, we show that in young animals, Draper is haploinsufficient with regard to activation of STAT92E transcription and glial phagocytic clearance of degenerating axons post injury. Collectively, our work highlights Draper-dependent signalling pathways as a molecular linchpin for proper glial immune responses across ages.

Finally, our results are also the first to suggest that WD is initiated more slowly in aged *Drosophila* olfactory nerves immediately after axotomy, as has been described for peripheral nerves in mammals^38^,^39^. The mechanisms of WD appear to be well conserved between flies and vertebrates^43^. As such, age-related changes in WD programmes and axon–glia signalling events may also be conserved across species. The specific molecules released by severed axons in adult *Drosophila* remain to be identified, but this *in vivo Drosophila* model offers a tractable platform to identify new axon-glia injury cues that are required to elicit glial responses and may be altered by normal ageing events.

In summary, our work highlights the importance of maintaining PI3K-dependent signalling and Draper in aged glia to maintain glial immunity and also suggests that cooperative input from both degenerating axons and local glia are required for efficient glial clearance of axonal debris in the senescent brain.

## Methods

### ORN axotomy assay

WD was induced in *Drosophila* ORN axons of the antennal nerve or the maxillary nerve by bilateral removal of the third antennal segment or the maxillary palp structures with forceps, respectively^15^,^16^. Unless shifted to 30 °C to inhibit Gal80^ts^ activity, flies were maintained at 22 °C–23 °C. For aged Draper rescue, we generated flies that carried *repo-Gal4*, a *UAS-Draper-I* transgene, and *OR85e-mCD8::GFP* to monitor clearance of maxillary palp ORNs. These flies also expressed a temperature-sensitive version of Gal80 (*tubulin-Gal80*^*ts*^) to temporally regulate the activity of Gal4. After raising flies at the permissive temperature (23 °C), we severed the maxillary nerves of young and aged flies, and then shifted flies to the restrictive temperature (30 °C) for 45 min to induce glial expression of Draper-I. Flies were returned to 23 °C and analysed 3 days later for clearance of axonal debris. Control flies were carried through the same temperature shift protocol. A similar protocol was used to induce expression of CA-PI3K for 7 days in glia of aged flies.

### Immunolabelling

Adult *Drosophila* heads were fixed (1 × PBS, 0.01% Triton X-100, 4% paraformaldehyde) at room temperature for 16 min. Samples were then washed 1 × 1 min and 2 × 5 min, while rocking in PBST × .01 (1 × PBS, 0.01% Triton X-100) at room temperatures. Fixed heads were kept on ice, while brains were dissected at room temperature in PBST × .01. Tissues were post fixed for 16 min in PBST × .1 (1 × PBS, 0.1% Triton X-100), washed 2 × 2 min and incubated overnight with primary antibodies in PBST × .1. The next day, brains were washed 4 × 30 min with PBST × .1 and incubated with secondary antibodies (in PBST × .1) for 2 h at room temperature. Brains were then washed 4 × 30 min with PBST × .1 and mounted on slides in VECTASHIELD mounting media (Vector Labs).

### Confocal microscopy and analysis

All brains were imaged on a Zeiss LSM 700 with a Zeiss × 40 1.4 numerical aperture oil-immersion plan-apochromatic lens. Brains within a single experiment (that is, those being directly compared for quantification) were whole mounted under a single #1.5 cover glass in VECTASHIELD. All brains in a given experiment were imaged on the same day with the same confocal microscope settings. Volocity 3D Image Analysis Software (Perkin Elmer) was used for fluorescence quantification and GraphPad Prism was used for statistical analysis. Quantification of GFP^+^ axonal debris from OR22a GFP-labelled axons and OR85e GFP-labelled glomeruli was performed on three-dimensional volumes segmented to the GFP signal in Volocity. Total intensity measurements were calculated and background fluorescence was subtracted. To quantify basal Draper levels in the cortex of adult brains, total intensity measurements were calculated in regions of interest (representative regions in young uninjured panel, white dotted line circle, Fig. 2e). See Fig. 2e panels for representative regions of interest (white dotted lines) selected to quantify Draper levels in glia responding to maxillary nerve and antennal nerve axotomy. Glial membrane recruitment to severed maxillary nerves was quantified by measuring total GFP fluorescence intensity in regions of interest similar to those outlined in Fig. 2e, maxillary palp injury (Supplementary Fig. 2)^15^,^16^. Glial membrane expansion after antennal nerve axotomy was quantified by measuring the thickness of GFP^+^ ensheathing glial membranes at several locations around each antennal lobe on single confocal slices at a consistent anterior depth of 4 μm into the brain.

### Antibodies

Primary antibodies were used at the following dilutions: chicken anti-GFP (#A10262 from ThermoFisher) at 1:1,000; mouse anti-Draper (hybridoma supernatants 8A1 and 5D14, now publicly available at Developmental Studies Hybridoma Bank) at 1:400 and mouse anti-Repo (8D12 from Developmental Studies Hybridoma Bank) at 1:20. Secondary antibodies (715-295-150 and 703-545-155 from Jackson Immunoresearch) were used at a dilution of 1:400.

### Western blot analysis

Whole adult heads were homogenized in 4 μl 1 × LB (Loading Buffer) per head. Protein lysate of four to five heads were loaded onto 4–20% Tris-Glycine gels (Lonza) and transferred to Immobilon-FL (Millipore). After transfer, total protein density per lane was measured using MemCode Reversible Protein Stain (Pierce ThermoFisher). Blots were probed with rabbit anti-Draper^18^ (1:1,000) or rabbit anti-Rpl32 (1:1,000, kind gift of Matthias Hentze) and incubated overnight at 4 °C, washed several times with 1 × PBS/0.01% Tween 20 and probed with appropriate fluorophore-conjugated antibodies secondary antibodies (713-625-147 and 711-655-152 from Jackson Immunoresearch) for 2 h at room temperature. Additional washes were performed with 1 × PBS/0.01% Tween 20 and a final wash in 1 × PBS. Total protein stain blots were imaged on G:BOX F3 Imaging System and analysed with ImageJ; fluorescent blots were imaged on Li-cor Odyssey CLx quantitative western blot imaging system and data were quantified using LiCor Image Studio software. Blot images in Figs 2 and 6 have been cropped for presentation. Full blot images are presented in Supplementary Fig. 6.

### *Drosophila* stocks

For all experiments, young flies were between 7 and 14 days old, whereas aged flies were between 56 and 63 days old. The following *Drosophila* genetic insertions were used in the paper: *OR85e-mCD8::GFP/CyO*^55^, *OR22a-Gal4/Cyo*^56^, *UAS-mCD8::GFP* (Bloomington Stock *5137*), *UAS-mCD8::GFP (*Bloomington Stock *5130*), *repo-Gal4* (ref. 15), *tubulin-Gal80*^*ts*^ (Bloomington Stock *7108*), *UAS-Draper-I/CyO*^16^, *UAS-caPi3K92E*^*caax*^ (Bloomington Stock 8294), *UAS-TOR-RNAi* (Bloomington Stock 33951), *UAS-mito-HA-GFP (*Bloomington Stock *8443*) and *10XSTAT92E-dGFP*^57^.

### Quantitative reverse transcriptase–PCR analysis

Total RNA from heads was extracted using Trizol LS (ThermoFisher), collected via RNA Clean & Concentrator-5 Kit (Zymo Research) and subject to DNAse digestion using Ambion DNA-free kit. Complementary DNA was prepared using qScript cDNA SuperMix kit (Quanta Biosciences). Total RNA was quantified using the Qubit RNA HS assay kit and Qubit Fluorometer, and equal amounts of RNA were added to cDNA synthesis reaction. Quantitative gene expression was carried out on an ABI 7,500 Fast Real-Time PCR machine using Taqman master mix (Applied Biosystems) and the following TaqMan assays: (i) RibosomalProtein L32 (Applied Biosystems premade assay Dm02151827_g1), (ii) Draper-I custom assay: F-primer, 5′-TGTGATCATGGTTACGGAGGAC-3′; R-primer, 5′-CAGCCGGGTGGGCAA-3′; probe, 5′-CGCCTGCGATATAA-3′.

### Polysome fractionation

Wild-type young and aged fly heads were manually homogenized with mortar and pestle in polysome lysis buffer (10 mM Tris-HCl, 150 mM NaCl, 5 mM MgCl_2_,0.5 mM dithiothreitol, 100 μG cycloheximide, EDTA-free protease inhibitor (Roche) and 40 U ml^−1^ Superase-in (Ambion)). The homogenate was centrifuged at 2,000 *g* for 10 min at 4 °C, to clear the cuticle debris, and 1% NP-40 was added to the supernatant before being incubated on ice for 10 min. Lysate was then cleared a second time by centrifugation at 16,000 *g* for 10 min at 4 °C. Lysates were layered onto a 10–60% sucrose gradient, centrifuged at 40,000 r.p.m. (SW-41Ti rotor) for 2 h at 4 °C and sampled on a Biocomp gradient station/Gilson fraction collector with constant monitoring of optical density at 254 nm^58^,^59^. Eleven 1 ml fractions were collected, each sample was spiked with 20 ng of luciferase RNA and total RNA was extracted from each fraction using the methods described above in quantitative reverse transcriptase–PCR analysis. Draper transcript levels in each fraction were normalized to Rpl32 (2^ΔCt^) and the percentage of Draper transcript in each fraction was adjusted using the Ct of luciferase, which reflected the RNA recovery rate in each fraction.

### Data availability

The authors declare that all data supporting the findings of this study are available within the article and its Supplementary Information files or from the corresponding author upon request.

## Additional information

**How to cite this article:** Purice, M. D. *et al*. Delayed glial clearance of degenerating axons in aged *Drosophila* is due to reduced PI3K/Draper activity. *Nat. Commun.* 7:12871 doi: 10.1038/ncomms12871 (2016).

## Supplementary Material

Supplementary Information Supplementary Figures 1-6.
