# Supplementary material for: Delayed glial clearance of degenerating axons in aged Drosophila is due to reduced PI3K/Draper activity
Source: Nat Commun. 2016 Sep 20;7:12871. doi: 10.1038/ncomms12871 (PMC5034330; doi:10.1038/ncomms12871)
Supplement: Supplementary Information — Supplementary Figures 1-6. [file ncomms12871-s1.pdf]

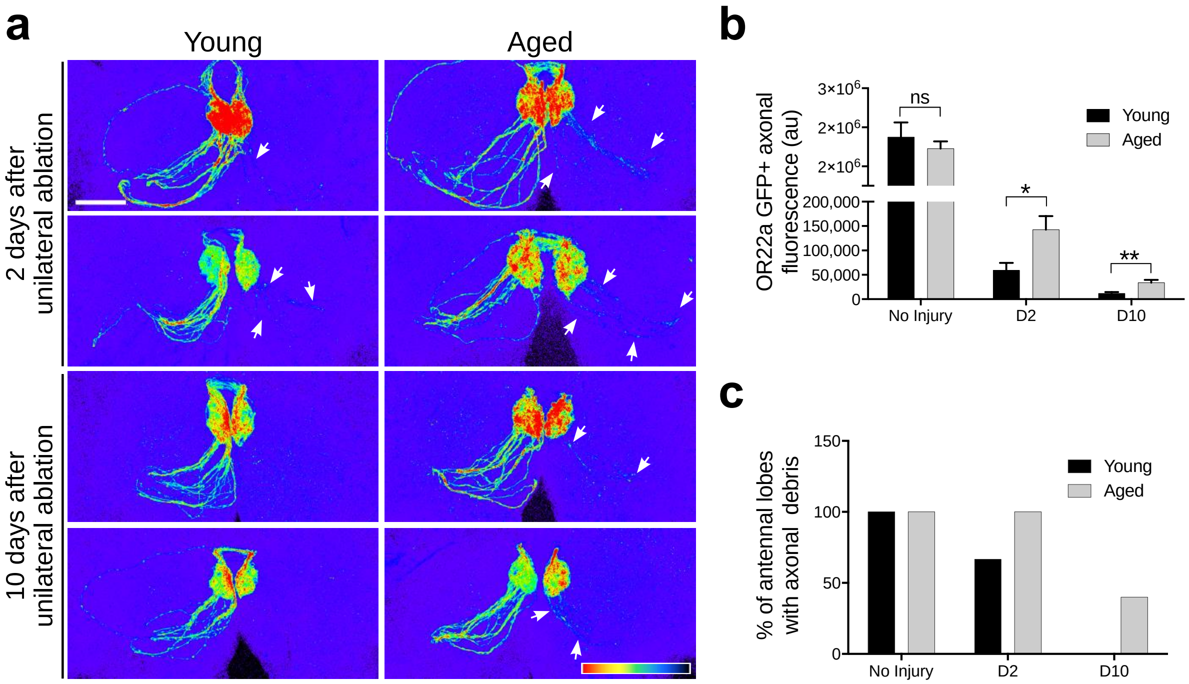

Supplementary Figure 1. Aged glia fail to efficiently clear degenerating axons in the adult *Drosophila* brain after unilateral antennal injury. (a) Two examples of GFP-labeled antennal ORN axon projections 2 or 10 days after unilateral antennal nerve axotomy in young and aged flies. Representative confocal projected stacks shown. White arrows point to persistant axonal debris in aged brains after antennal nerve injury. Images have been converted to heat scale (red to black, high to low fluorescence respectively) to show that GFP levels are comparable between young and aged animals. (b) Quantification of raw GFP fluorescence in OR22 axons from experiment shown in (a); mean ± s.e.m. plotted; $^*P < 0.05$, $^{**}P < 0.01$, unpaired t test. N≥7 antennal lobes. (c) Percentage of antennal lobes containing visible tracts of antennal GFP+ axons after unilateral injury.
Scale bar = 30 um. Genotype: w^{1118};OR22a-Gal4, UAS mCD8::GFP/+.

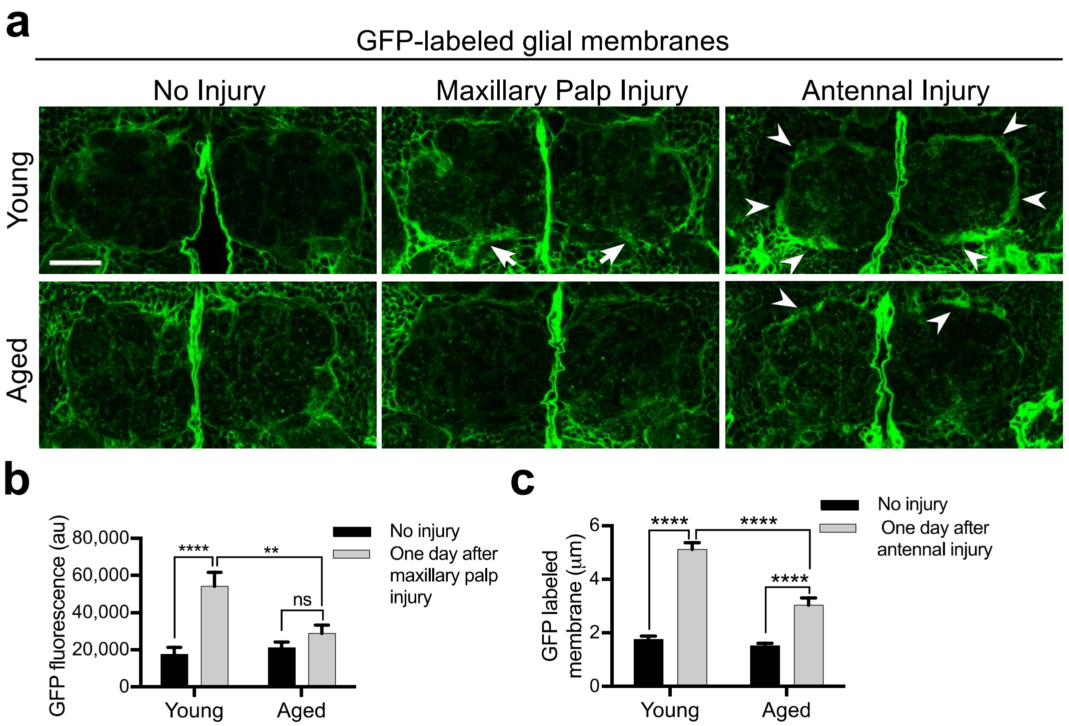

Supplementary Figure 2. Glial membrane expansion to sites of axon injury is attenuated in aged flies. (a) Glial membranes were labeled in vivo with membrane-tethered GFP (repo-Gal4, UAS-mCD8::GFP). Robust accumulation of glial membranes was observed on degenerating axons in young flies after maxillary (arrows) or antennal nerve (arrowheads) axotomy. Representative confocal slices shown. (b) Quantification of GFP+ fluorescence on degenerating maxillary palp axons one day after axotomy; mean ± s.e.m. plotted; **$P<0.01$, ****$P < 0.0001$. (c) Quantification of GFP^+ glial membrane expansion after antennal nerve injury; mean ± s.e.m. plotted; ****$P < 0.0001$. N ≥20 antennal lobes for each time point. Scale bar = 30 um. Two-way ANOVA with Sidak post hoc test was performed. Genotype: w^{1118};repo-Gal4, UAS-mCD8::GFP/TM3.

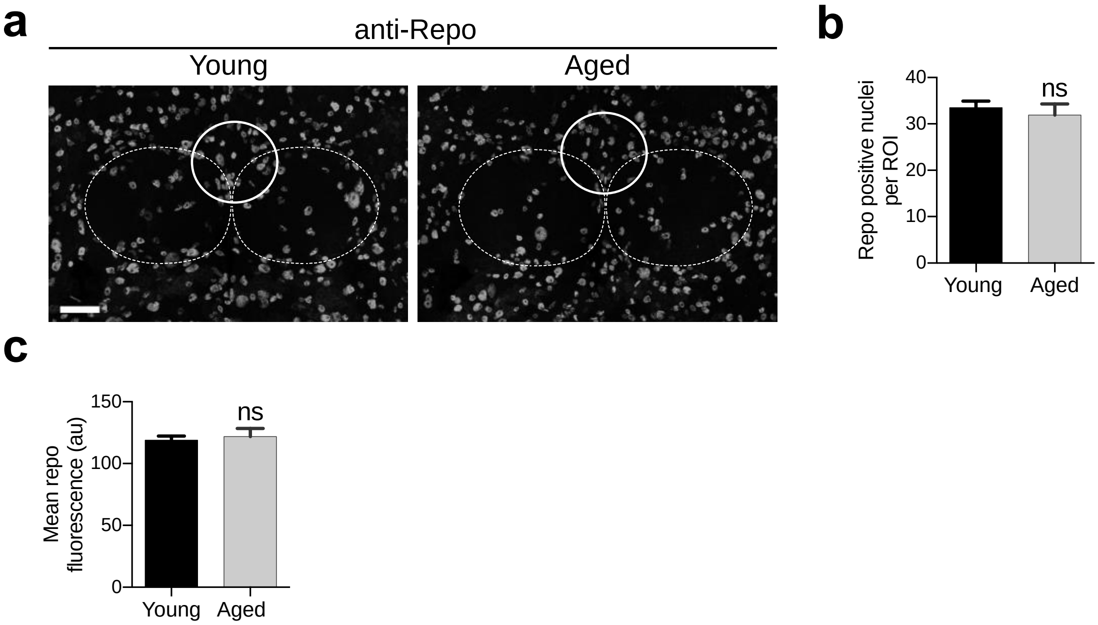

Supplementary Figure 3. Glial cell numbers in the antennal lobe region do not change with age. (a) Representative compressed 20 um confocal Z-stack images of young and aged brains immunostained for the glial-specific nuclear transcription factor Repo. Antennal lobes outlined with dotted line. (b) Counts of Repo+ nuclei in the central brain region adjacent to the antennal lobes. (c) Quantification of Repo fluorescence in the cortex of young and aged brains.
(b) and (c) ROI used for counting and fluorescence measurements shown in solid white circle. N=15; mean ± s.e.m. plotted; n.s.= not significant from unpaired t-test. Scale bar = 20 um.
Genotype: w^{1118};repo-Gal4, UAS-mCD8::GFP/TM3.

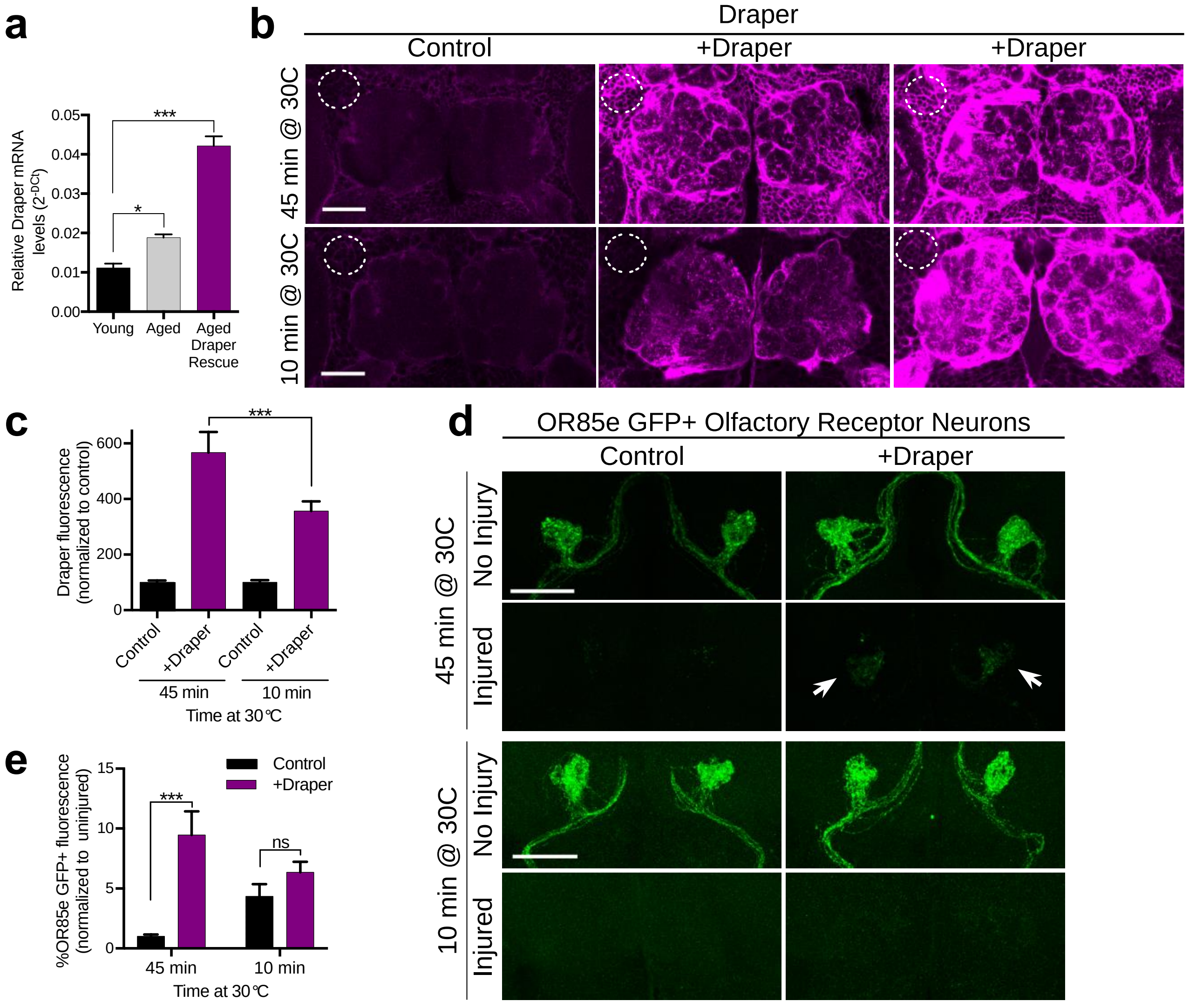

Supplementary Figure 4. Effects of GAL4/UAS-driven Draper-I on *draper-I* transcript levels and clearance function. (a) Representative Q-PCR of *draper-I* in young, aged and aged Draper rescue brains shown in Figure 4. mean ± s.e.m. plotted, *P<0.05, ***P < 0.001; One-way ANOVA with Bonferroni post hoc test. N=3 biological replicates/group. (b-e) The Gal4/Gal80ts system was used to acutely upregulate Draper-I expression in glia of young flies for either 10 minutes or 45 minutes at the time of maxillary nerve injury, as described in Fig 4a. (b) Representative Draper immunostainings of control and UAS-Draper-I overexpression flies shifted to 30°C for either 45 minutes or 10 minutes (two examples of each shown). White dotted outline on single confocal slice images shows representative ROI used for quantification of basal Draper in (c). (c) Quantification of cortical Draper fluorescence shown in (b) normalized to controllevels. mean ± s.e.m. plotted; ***P < 0.001, One-way ANOVA with Sidak post hoc test; N=20 hemi brains. (d) Representative confocal Z-stack projections of OR85e GFP-labeled axons. Some persistent axonal debris remained in the antennal lobes 3 days after injury in flies treated with 45 minute 30°C pulse to raise Draper levels (arrows) but not following a 10 minute pulse. (e) Quantification of GFP+ axonal debris in experiment shown in (d). Mean ± s.e.m. plotted; ns=not significant, ***P<0.001; unpaired t test. N ≥24 antennal lobes. Scale bars: 30 um. Genotypes: Control Young and Aged = w^{1118};OR85e-mCD8::GFP, tubulin-Gal80ts/+; repo-Gal4/+. Aged Draper Rescue = w^{1118};OR85e-mCD8::GFP, tubulin-Gal80ts/UAS-Draper-I; repo-Gal4/+.

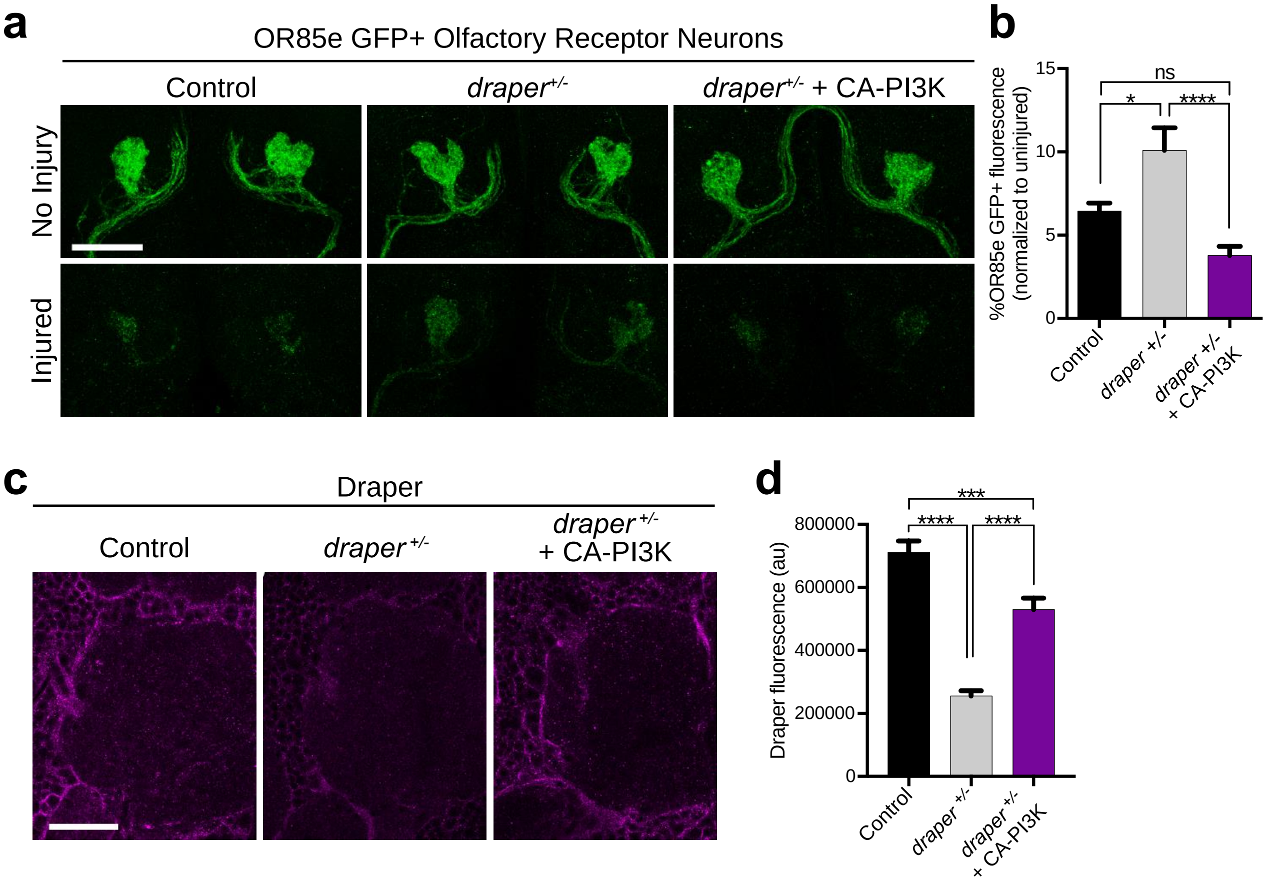

Supplementary Figure 5. Expression of CA-PI3K in draper heterozygotes rescues glial clearance of axonal debris. (a) OR85e GFP+ axonal projections in uninjured flies versus 2 days after maxillary nerve axotomy. Expression of CA-PI3K in glia lacking a copy of Draper restored normal clearance of OR85e axonal debris. Representative Z-stack projections shown. (b) Quantification of axonal clearance in (a); mean ± s.e.m. plotted; ns = not significant, *P<0.05, ****P < 0.0001. One-way ANOVA with Sidak post hoc test. N ≥18 antennal lobes. (c) Representative images of Draper immunostainings. (d) Quantification of Draper fluorescence in the cortex of animals shown in (c); mean ± s.e.m. plotted; ***P<0.001, ****P < 0.0001, One-way ANOVA with Sidak post hoc test; N ≥19 hemi brains. All scale bars = 30 um. Genotypes: Control = w^{1118};OR85e-mCD8::GFP, tubulin-Gal80^{ts}/+; repo-Gal4/+. Draper+/- = w^{1118};OR85e-mCD8::GFP, tubulin-Gal80^{ts}/+; repo-Gal4/ draperΔ5. Draper+/- + CA-PI3k = UAS-PI3K92eCAAX/ w^{1118};OR85e-mCD8::GFP, tubulin-Gal80ts/+; repo-Gal4/draperΔ5.

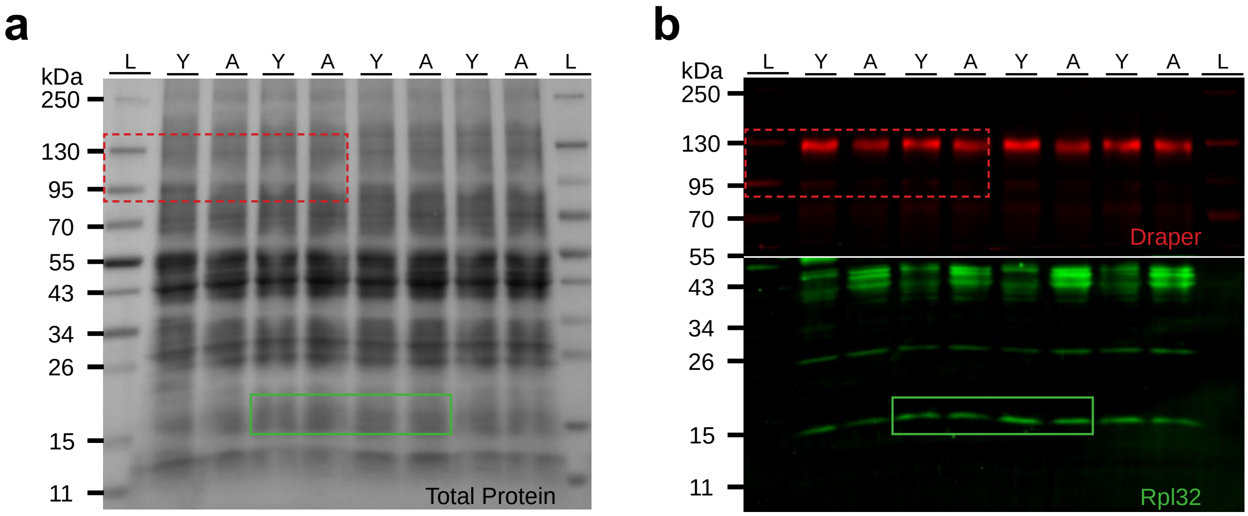

Supplementary Figure 6: Uncropped blots for Westerns shown in Figures 2a and 6i. (a) Total protein blot displaying four biological replicates for young (Y) and aged (A) samples. (b) Fluorescence image of the blot displayed in (a) probed for Draper (red, top half) and Rpl32 (green, bottom half). The red dotted boxes highlight the cropped areas in Fig 2a; green boxes highlight the cropped areas shown in Fig 6i.
